# The Virome and Its Major Component, Anellovirus, a Convoluted System Molding Human Immune Defenses and Possibly Affecting the Development of Asthma and Respiratory Diseases in Childhood

**DOI:** 10.3389/fmicb.2018.00686

**Published:** 2018-04-10

**Authors:** Giulia Freer, Fabrizio Maggi, Massimo Pifferi, Maria E. Di Cicco, Diego G. Peroni, Mauro Pistello

**Affiliations:** ^1^Retrovirus Center, Department of Translational Research, University of Pisa, Pisa, Italy; ^2^Virology Unit, University Hospital of Pisa, Pisa, Italy; ^3^Department of Clinical and Experimental Medicine, Section of Pediatrics, University of Pisa, Pisa, Italy

**Keywords:** virome, microbiome, anelloviruses, torque teno virus, asthma, respiratory diseases, wheezing

## Abstract

The microbiome, a thriving and complex microbial community colonizing the human body, has a broad impact on human health. Colonization is a continuous process that starts very early in life and occurs thanks to shrewd strategies microbes have evolved to tackle a convoluted array of anatomical, physiological, and functional barriers of the human body. Cumulative evidence shows that viruses are part of the microbiome. This part, called virome, has a dynamic composition that reflects what we eat, how and where we live, what we do, our genetic background, and other unpredictable variables. Thus, the virome plays a chief role in shaping innate and adaptive host immune defenses. Imbalance of normal microbial flora is thought to trigger or exacerbate many acute and chronic disorders. A compelling example can be found in the respiratory apparatus, where early-life viral infections are major determinants for the development of allergic diseases, like asthma, and other non-transmissible diseases. In this review, we focus on the virome and, particularly, on *Anelloviridae*, a recently discovered virus family. Anelloviruses are major components of the virome, present in most, if not all, human beings, where they are acquired early in life and replicate persistently without causing apparent disease. We will discuss how modulation of innate and adaptive immune systems by Anelloviruses can influence the development of respiratory diseases in childhood and provide evidence for the use of Anelloviruses as useful and practical molecular markers to monitor inflammatory processes and immune system competence.

## Introduction

At birth, both the digestive system and the airways are immediately exploited as portals of entry by a number of microbes, most of which are likely to persist and become part of the so-called “microbiome.” This is a community of microorganisms that live on the human body without apparently affecting health (Whipps and Karen Lewis, 1988; Hooper et al., 2012; Tremaroli and Bäckhed, 2012). It has long been known that the microbiome is beneficial to hosts in a number of ways, and, in recent years, its interaction with the immune system has even been recognized as fundamental for immune system maturation, reactivity to specific antigens and development of tolerance (Scharschmidt et al., 2015; Ignacio et al., 2016; Kim et al., 2016). The microbiome, from this point of view, tunes immunity by acting as a constant source of stimuli (Belkaid and Hand, 2014; Belkaid and Segre, 2014).

Recently, with the advent of high throughput sequencing methods, the diversity of the microbiome inhabiting gut, lung, skin, and even blood in physiological conditions has been found to be much larger than first thought. In particular, a constantly fluctuating population of viruses have joined the list of infectious agents that are now considered part of the microbiome in several body sites (Blaser and Valentine, 2008; Shulman and Davidson, 2017). Very recent work has estimated that roughly 45% of mammalian viruses can be detected in healthy humans (Olival et al., 2017).

Most initial interactions between hosts and viruses are governed by the innate immune system, that prevents colonizing infectious agents from spreading systemically and maintains mucosal homeostasis (Medzhitov and Janeway, 2002; Lamkanfi and Dixit, 2011). Activation of the innate immune responses triggers a cascade reaction that results in secretion of cytokines and chemokines, and often engages different cells to control invasion (Freer et al., 2017). Following recognition of specific microbial, viral and damage stimuli, intracellular multiprotein complexes called inflammasomes assemble and induce downstream immune responses to specific pathogens. The effects of turning on immunity generally protects against pathogen invasion, but reactions to harmless antigens may lead to the establishment of disease in predisposed individuals. In this review, we discuss the multiple effects of the virome on host health, with special reference to Anelloviruses.

## The Human Virome

Although viruses have long been considered “bad news in a protein coat” (Medawar and Medawar, 1983), many novel viruses are found to replicate in healthy individuals. So far, roughly 220 viruses are known to infect humans and only about half are pathogens (Parker, 2016). Truly apathogenic viruses can be grossly divided in viruses infecting bacteria, integrating into human chromosomes as endogenous retroelements, and persisting indefinitely. They are referred to as “commensal” viruses that are part of the virome without an apparent clinical outcome (Rascovan et al., 2016). Many viruses that infect humans may even have a beneficial role (Phan et al., 2016): in animal models, resident intestinal viruses were shown to reduce intestinal inflammation by inducing interferon (IFN)-β, secreted mainly by plasmacytoid dendritic cells (DCs) (Yang et al., 2016), or by providing resistance to infection by bacterial pathogens (Barton et al., 2007).

The number of apathogenic viruses includes many genera detected in various tissues of healthy people, especially infants (Allander et al., 2005; Wang et al., 2016; Moustafa et al., 2017). What role they play in human physiology is still unknown, although they are currently hypothesized to alter disease susceptibility. This is suggested by many epidemiological observations and findings in animal models (Roossinck, 2011; Virgin, 2014).

Resident viruses influence the immune system helping it to develop properly, similarly to bacterial microbiome. Indeed, Cadwell demonstrated that mouse norovirus, a commensal relative of a human pathogen, restored intestinal morphology and immunological functions in germ-free newborn mice, where it is normally perturbed (Cadwell, 2015). On the other hand, the immune system has been recently demonstrated to control virome expansion, similarly to bacteria: HIV-infected patients exhibited low peripheral CD4^+^ T cell counts and dramatic expansion of enteric virome adenovirus titers, possibly contributing to AIDS-associated enteropathy and disease progression. These findings suggest that virome expansion is linked to the pathogenesis of AIDS and highlights the role of the immune system in controlling viral populations in the intestine (Monaco et al., 2016). In addition, enteric viral communities have been found to change during HIV infection and raises in Anelloviridae and other virus titers have been associated to increased pathology (Gootenberg et al., 2017).

### Anelloviruses and TTV

A group of viruses discovered in 1997 (Nishizawa et al., 1997; Okamoto et al., 1998), now called Anelloviruses (AV), represents about 70% of total viruses detected in blood and in most tissues and organs (De Vlaminck et al., 2013). Their prototype, presently named torquetenovirus (TTV), is one of a vast spectrum of viral agents with similar genomes, like torquetenominivirus (Takahashi et al., 2000) and torquetenomidivirus (Ninomiya et al., 2007), both of which have smaller genomes than TTV. All these viruses are classified in the newly established family Anelloviridae (from *anellus*, Latin for ring, for their circular genome). AV are characterized by a small (2.2 to 3.7 kb), single stranded DNA (ssDNA) circular genome, which makes AV the genetically simplest of all known replication-competent animal viruses. In addition, they are extremely diverse genetically, more than any other viral family. They all lead to persistent, possibly life-long infections and they can be detected at very high levels in blood and in practically all tissues of almost 100% of people worldwide. Different genetic forms are found in a large proportion of individuals regardless of age, socio-economical standing and health conditions, being acquired very soon after birth or even prenatally (Maggi and Bendinelli, 2010).

No specific pathogenic effect has so far been pinpointed to any AV, although similarity of human *Anelloviridae* to avian ones suggests that their pathogenicity might be underestimated (Davidson and Shulman, 2008). Increased viremia levels of AV have been found in immune suppressed individuals and in subjects with inflammatory diseases, suggesting that they are normally kept under immunological control, but may contribute to maintain the background level of inflammation chronically elevated in the body (Maggi et al., 2004; Li et al., 2013; Young et al., 2015; Abbas et al., 2016).

### How TTV Interacts and Modulates Host Defenses

TTV interacts with many pathogen-associated molecular pattern (PAMP) receptors (PRR) that fuel immune and inflammatory responses (Zheng et al., 2007; Rocchi et al., 2009; Kincaid et al., 2013). *In vitro* studies show that TTV ORF2 protein suppressed the activity of NFκB, crucial for the expression of many genes connected to inflammation. ORF2 protein of TTV is able to influence the activity of NFκB by inhibiting its translocation to the cell nucleus and, consequently, its ability to activate transcription of genes, such as IL-6, -8, and cyclo-oxygenase-2 (Zheng et al., 2007).

In addition, the genome of TTV and its replication intermediates may stimulate TLRs in infected cells and consequently synthesis of pro-inflammatory molecules. Unmethylated heterodimers of guanosine and cytosine (CpGs) in bacterial and viral DNA are absent in mammalian DNA and therefore seen as molecular signatures of foreign DNA. The importance of these molecules as PAMPs is demonstrated by the fact that one PPR, namely toll-like receptor (TLR)-9, is specialized to detect CpGs. Depending on the number or nucleotides flanking CpGs, it triggers production of inflammatory cytokines, such as IFN-α, Interleukin (IL)-6, and IL-12, or, alternatively, it may generate an inhibitory signal (Krieg, 2002). Both stimulatory and inhibitory CpGs are present in DNA of TTV and in most microbes, and their relative frequency may differ considerably, even within strains of the same species, thus probably influencing the way they interact and stimulate TLR-9. For instance, we have found that TTV genogroup 4, detected at higher levels in pediatric patients with bronchopneumonia compared to those with milder acute respiratory diseases (ARDs) (Maggi et al., 2003), was rich in stimulatory CpGs and activated TLR-9 in mouse spleen cells *in vitro*, causing abundant production of pro-inflammatory cytokines (Zheng et al., 2007; Rocchi et al., 2009).

MicroRNAs (miRNA) are ∼22 nt small, single-stranded, non-coding RNAs produced by hosts and pathogens. They are potent modulators of pathogen recognition and host defense in a vast array of cellular metabolic pathways. As regards microbe–host interaction, cellular miRNAs seem to modulate immune responses and inflammation and to play a direct antiviral role by blocking translation of viral genes, counteracting block of apoptosis and persistent replication. Very recent work shows that miRNA can polarize macrophages toward allergic reactions in animal models (Zhou et al., 2017). Their role in inflammation is probably very complex, since they may both up- and down-regulate inflammation in several diseases, including asthma (Dissanayake and Inoue, 2016). Viruses, including small ones like TTV, encode their own miRNAs that cooperate with viral proteins to regulate the expression of viral genes, replication, pathogenesis and immune evasion, and the whole process of virus-related inflammation (Kincaid and Sullivan, 2012; Cullen, 2013; Sorel and Dewals, 2016). Of note, both cellular and viral miRNAs have been found to transmit information to distant cells by circulating within plasma exosomes.

**FIGURE 1:**
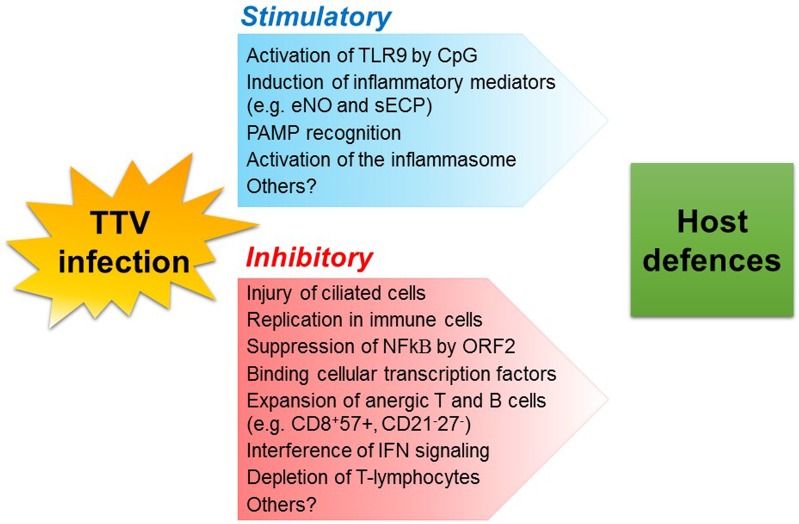
Possible mechanisms whereby TTV infection may modulate host defenses. TLR, toll-like receptor; CpG, unmethylated heterodimers of guanosine and cytosine; eNO, exhaled nitric oxide; sECP, secreted eosinophil cationic protein; PAMP, pathogen-associated molecular pattern.

Interestingly, TTV was also found to encode *in vivo* miRNAs possibly involved in viral immune evasion and that could be involved in the regulation of IFN signaling (Kincaid et al., 2013). Different TTV species have been shown to encode miRNAs and cause these molecules to be found as plasma exosomes in many infected individuals. Production and release was not correlated with virus replication, as monitored by measuring TTV viremia levels. Notably, TTV miRNA profiles differed depending on patients’ health status; the type of miRNAs produced also differed within sick patients (Vignolini et al., 2016). Role and significance of TTV miRNAs are not yet understood and warrant further studies. An overview of the mechanisms exploited by TTV to stimulate or soothe host innate and adaptive defenses is shown in **Figure 1**.

### Role of Microbial Exposure and Viral Infections in Wheezing and Asthma Development

Asthma is a multifactorial inflammatory disease of the lower airways, caused by environmental and genetic factors. The disease incidence seems to be increasing especially in industrialized countries (Ellwood et al., 2017). A long-standing theory, the hygiene hypothesis, suggests that insufficient interaction with microbes in early life leads to the development of allergic reactions (Liu, 2015). Significant differences in the prevalence of asthma were found between Amish and Hutterite schoolchildren, despite similar genetic ancestries and lifestyles. As compared with the Hutterites, the Amish practice traditional farming and are exposed to an environment rich in microbes. The significantly lower rates of asthma and the distinct immune profiles in the Amish suggest that environment and sustained microbial exposure have profound effects on innate immunity (Stein et al., 2016). Further data generated in an experimental model of asthma support this notion by showing that the protective effect of the Amish environment requires the activation of innate immune signaling.

On the other hand, there is a consensus on the notion that early respiratory viral and bacterial infections are potent triggers of wheezing-related disorders and development of asthma later in life (Lemanske, 2004; Gern et al., 2005). Growing evidence indicates that respiratory viral infections, especially when acquired in early life may provide the stimulus to inflammasomes assembly, and prime immature DCs toward a Th2 response that, eventually, may sensitize genetically predisposed individuals to local allergens (Holgate, 2011; Lee et al., 2014).

Virus and microorganisms in general may act through several PRRs including TLRs (TLRs and TLR-9 in particular) on DCs. Virus infections have been shown to modulate the responses of TLRs and miRNAs on the balance of T cells toward Th1, Th2, Th17, or T-regulatory (Treg) subtypes in respiratory airways (Durrani et al., 2012; Holt and Sly, 2012). Microbial products may in turn bind TLRs on airway epithelial cells, leading to the release of the IL-7-like cytokines and thymic stromal lymphopoietin. They may also interact with TLRs on DCs and upregulate the expression of costimulatory molecules to enhance Th2 polarization, also activating mast cells for Th2 cytokine production (de Heer et al., 2004; Troy and Bosco, 2016). Recently, the role of respiratory syncytial virus (RSV) and its impact on bronchiolitis at the time of infection and respiratory morbidity later in life has been revisited (Rossi and Colin, 2017). It has been shown, although controversially, that patients with RSV infection receiving hospital-based care have a higher incidence of asthma and reduced pulmonary function in childhood and in adolescence (Sigurs et al., 2010). In addition, large-scale use of molecular diagnostic techniques pinpointed human rhinovirus (HRV) to infant wheezing and asthma development (Song, 2016). Indeed, HRV has been isolated in 90% of children with asthma exacerbations and found closely connected to hospitalization risk in cohort studies (Bizzintino et al., 2010; Foxman and Iwasaki, 2011). Further evidence on the role of HRV infections during infancy in asthma development has been found: the Childhood Origin of Asthma (COAST) cohort study showed that at least one HRV infection during infancy was the most significant risk factor for persistent wheezing at the age of 3 years (Lemanske et al., 2005). Also, having an HRV wheezing episode in the first 3 years of life was a strong risk factor for asthma not only in childhood (Hyvärinen et al., 2005; Jackson et al., 2015), but also in adolescence (Rubner et al., 2017). In fact, in a high-risk birth cohort, HRV wheezing, associated with early life aeroallergen sensitization, had additive effects on asthma risk at adolescence (Rubner et al., 2017). Other detrimental effects have been associated to various respiratory viruses, such as metapneumovirus and bocavirus (Camargo et al., 2008; Söderlund-Venermo et al., 2009; Rudd et al., 2017). Indeed, atopy predisposition may be the individual driving factor involved in promoting asthma development via interaction with HRV, and possibly other respiratory viral infections, in infancy (Song, 2016). Allergic sensitizations and viral infections may in turn skew immune responses to produce Th2 cytokines that may at the same time amplify allergic inflammation and reduce the host antiviral responses, resulting in increased viral replication and cellular damage (Xatzipsalti et al., 2008).

### AV and Respiratory Allergy

Although TTV has been investigated to a reasonable extent, its role on asthma is far from clear. Previous studies of our group have shown that presence or viremia levels of TTV were significantly associated with ARDs in pediatric patients and that children with bronchopneumonia (BP) have considerably higher TTV loads than do children with milder respiratory disease. Further, high TTV loads correlate with a decrease in circulating CD3^+^ and CD4^+^ T cells, an increment in B cells, and increased activity of eosinophils, again emphasizing the immunomodulatory activity of TTV (Maggi et al., 2004; Pifferi et al., 2005). In another study, a positive association was found between nasal TTV loads and levels of eosinophil cationic protein, a marker of bronchial inflammation. These markers were found particularly elevated in the children with asthma who had moderately to severely compromised spirometric indices. This study was the first performed in children with asthma and suggested that TTV might be a contributing factor in lung impairment (Pifferi et al., 2005).

Concerning a mechanistic role of TTV in respiratory dysfunction, it has been postulated that this virus, either alone or synergistically with other viruses, may act as an enhancer of inflammation systemically or at specific body sites such as upper and lower airways (Maggi and Bendinelli, 2009). One possible way can be envisioned via high amounts of immune complexes that form following TTV replication in blood. In infants with ARD, the airways were shown to be the sites of primary infection and continual replication by TTV, with higher viral loads in patients with more severe lower respiratory tract infections (Maggi et al., 2003). Furthermore, TTV may worsen the extent and the severity of the inflammatory response due to allergens, if sensitization is present in the subject. This hypothesis is supported, in children with ARD, by the positive correlation between TTV loads and serum concentration of eosinophil cationic protein (Maggi et al., 2004), and of exhaled nitric oxide, a sensitive marker of airway inflammation in asthmatic children (Li et al., 2013). In another study, we were able to demonstrate a high prevalence of TTV infections in children with bronchiectasis, a chronic respiratory disorder associated with several invalidating airway diseases: indeed, strong correlation between TTV loads and airflow limitation within the more peripheral airways was found, as well as between severity of the disorder and limitation of the lung function (Pifferi et al., 2006).

## Conclusion

Most viral infections elicit robust immune responses but viral clearance is not always obtained. Consequently, there must be unidentified factors/conditions that determine a tolerogenic status toward non-pathogenic viruses, and strong immune opposition against pathogenic ones. Tolerance may depend on host genetic, life-style and environmental factors, or alternatively on the ability of “commensal” viruses not to stir up inflammasomes and innate immune effectors.

Increasing evidence shows that the virome is actually beneficial to the host, who seems to tolerate “commensal” viruses, although they replicate and circulate among individuals. AV infect and persist in nearly all mammals and represent a large body of the human virome. They continuously replicate with no overt damage to the host and, therefore, are a good example of commensal viruses in this respect. Several clinical studies suggest that TTV plays a role in the development and/or exacerbation of respiratory diseases in childhood. Although further studies are warranted to draw firm conclusions, the virome and AV are one the best examples of a virus–host relationship. Its understanding will help clarify the role of viruses in shaping human immune defenses and perhaps contribute to their derangement.

## Author Contributions

GF, FM, DP, and MauP contributed to the elaboration of this mini review. MasP and MDC performed TTV studies in infants and made some unpublished results available. All authors read and approved the final manuscript.

## Conflict of Interest Statement

The authors declare that the research was conducted in the absence of any commercial or financial relationships that could be construed as a potential conflict of interest.
